# *Salmonella* Typhimurium expressing chromosomally integrated *Schistosoma mansoni* Cathepsin B protects against schistosomiasis in mice

**DOI:** 10.1038/s41541-023-00599-w

**Published:** 2023-02-27

**Authors:** Adam S. Hassan, Sébastien Houle, Lydia Labrie, Dilhan J. Perera, Charles M. Dozois, Brian J. Ward, Momar Ndao

**Affiliations:** 1 Department of Microbiology & Immunology, McGill University, Montreal, QC Canada; 2 Infectious Diseases and Immunity in Global Health (IDIGH), Research Institute of the McGill University Health Centre, Montreal, QC Canada; 3 INRS-Centre Armand-Frappier Santé Biotechnologie, Laval, QC Canada; 4 Division of Experimental Medicine, McGill University, Montreal, QC Canada

**Keywords:** Live attenuated vaccines, Parasitic infection

## Abstract

Schistosomiasis threatens hundreds of millions of people worldwide. The larval stage of *Schistosoma mansoni* migrates through the lung and adult worms reside adjacent to the colonic mucosa. Several candidate vaccines are in preclinical development, but none is designed to elicit both systemic and mucosal responses. We have repurposed an attenuated *Salmonella enterica* Typhimurium strain (YS1646) to express Cathepsin B (CatB), a digestive enzyme important for the juvenile and adult stages of the *S. mansoni* life cycle. Previous studies have demonstrated the prophylactic and therapeutic efficacy of our plasmid-based vaccine. Here, we have generated chromosomally integrated (CI) YS1646 strains that express CatB to produce a viable candidate vaccine for eventual human use (stability, no antibiotic resistance). 6–8-week-old C57BL/6 mice were vaccinated in a multimodal oral (PO) and intramuscular (IM) regimen, and then sacrificed 3 weeks later. The PO + IM group had significantly higher anti-CatB IgG titers with greater avidity and mounted significant intestinal anti-CatB IgA responses compared to PBS control mice (all *P* < 0.0001). Multimodal vaccination generated balanced T_H_1/T_H_2 humoral and cellular immune responses. Production of IFNγ by both CD4^+^ and CD8^+^ T cells was confirmed by flow cytometry (*P* < 0.0001 & *P* < 0.01). Multimodal vaccination reduced worm burden by 80.4%, hepatic egg counts by 75.2%, and intestinal egg burden by 78.4% (all *P* < 0.0001). A stable and safe vaccine that has both prophylactic and therapeutic activity would be ideal for use in conjunction with praziquantel mass treatment campaigns.

## Introduction

Trematode worms of the *Schistosoma* genus are responsible for schistosomiasis, a freshwater-borne disease that affects over 250 million people worldwide. Approximately 800 million people are at risk of infection caused primarily by three human *Schistosoma* spp.: *S. mansoni*, *S. haematobium*, and *S. japonicum*. As the most widespread, *S. mansoni* is prevalent in Sub-Saharan Africa, South and Central America, the Middle East, and parts of the Caribbean^1^. Although school-aged children are most affected, all age groups can suffer from schistosomiasis pathology, including debilitating disease^2,3^.

The oral anthelmintic, praziquantel (PZQ), is the cornerstone of schistosomiasis control in endemic regions. National or regional deworming campaigns typically deliver a single dose of 40 mg/kg and cure rates are reported to vary between 60–90%^4^. However, overreliance on PZQ has led to concerns about drug resistance which has been observed experimentally in both in vitro and in vivo studies^5,6^. While periodic deworming and vector management are useful to control schistosomiasis, vaccines will likely be necessary to achieve elimination^7^. Several candidate vaccines are in preclinical development, and few have entered clinical trials in recent years^7^. Most of these candidate vaccines are based on repeated intramuscular injection of one or more recombinant proteins with an adjuvant. In contrast, our group has established a vaccination platform based on a live attenuated *Salmonella enterica* serovar Typhimurium strain, YS1646, that can be administered orally^8,9^. Our strategy also differs from most others by targeting a key digestive enzyme of the parasite, Cathepsin B (CatB), rather than external tegumental proteins^10^. This novel approach results in significant reductions in parasite burden when used either prophylactically in a 3-week prime-boost regimen or therapeutically in a shortened 5-day schedule in chronically infected mice.

Previous studies with our recombinant *Salmonella* strains have used plasmid-based expression of CatB^8,9^, which would be unacceptable for human use. These strains are intrinsically unstable since bacteria can shed plasmids during cell division in an environment that does not select for their retention^11^. Furthermore, retention of plasmids typically involves the use of one or more antibiotic resistance genes. The inclusion of such resistance genes on a mobile genetic element is clearly inappropriate for human use due to the risk of spreading resistance to enteric bacteria. As a result, chromosomal integration of the antigen construct is crucial to the advancement of these vaccine candidates to the clinic. An obvious concern with chromosomal integration is a sharp reduction in the CatB copy number. In a plasmid-based system, each recombinant *Salmonella* may contain 30 copies or more of the CatB gene, while chromosomal integration typically introduces only a single copy of the gene.

In the current work, we report the construction of a chromosomally integrated, single-copy CatB recombinant YS1646 *Salmonella* Typhimurium strain. Vaccination with this novel vector resulted in significant reductions in parasite burden in a murine model. Multimodal immunization led to robust humoral and cellular immune responses with a balanced T_H_1/T_H_2 profile. To our knowledge, this is the first report of a chromosomally integrated *Salmonella*-vectored vaccine for schistosomiasis.

## Results

### Chromosomal integration of vaccine constructs in *Salmonella* Typhimurium YS1646

**Fig. 1:**
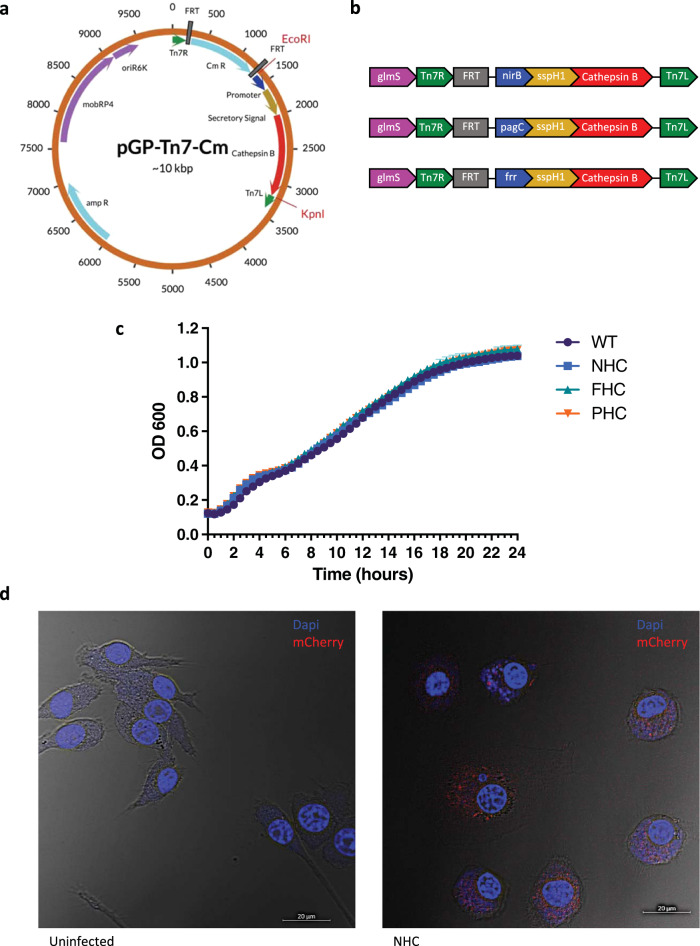
Chromosomal integration. Map of the pGP-Tn7-Cm plasmid designed to include a promoter, secretory signal, and CatB antigen. Insertion occurred between the EcoRI and KpnI restriction sites (**a**). Linear integrated sequences for each YS1646 construct following FLP-FRT recombination to remove the chloramphenicol resistance gene (**b**). Growth curves of WT and recombinant YS1646 strains measured by absorbance at OD_600_ over 24 h (*n* = 4 readings per 30 min interval) (**c**). Confocal microscopy images of uninfected and infected RAW264.7 cells at 63X magnification. DAPI nuclear stain is represented in blue and mCherry expression is shown in red (**d**). The scale is set to 20 μm.

We first generated plasmids bearing different promoters (*nirB*, *pagC*, *frr*) with the *Salmonella* SspH1 secretion signal fused to the CatB peptide sequence cloned into the pGP-Tn7-Cm vector^11^ (Fig. 1a). A chloramphenicol resistance gene, flanked by two FRT sites, within the Tn7 transposon arms, Tn7L and Tn7R, was used for selection of colonies where Tn7-mediated integration may have occurred. Constructs were inserted using EcoRI and KpnI cut sites. Tn7-mediated integration occurs at the *att*Tn7 site of *S*. Typhimurium downstream of the *glmS* gene.

By FLP-FRT recombination, we removed the chloramphenicol resistance cassette from the region that integrated at the *att*Tn7 site to generate three candidate vaccine vectors in which expression was driven by promoters *nirB* (NHC)*, pagC* (PHC), or *frr* (FHC) (Fig. 1b). Integration of these constructs occurred downstream of the *glmS* gene in YS1646 *S*. Typhimurium. *Salmonella* promoters *nirB* and *pagC* are active intracellularly upon induction by host oxygen and magnesium levels, whereas the *frr* promoter is constitutively active. Prior to the removal of the Cm cassette as part of an intermediate validation step, recombinant antigen expression was confirmed in strains containing the *nirB* and *pagC* constructs, but not the *frr* promoter-based construct. All three chromosomal integrations had no obvious impact on bacterial fitness and growth rates were similar to parental strain YS1646 (Fig. 1c).

The nirB_SspH1_CatB (NHC) construct was selected for further assessment in vitro and in vivo. Using a RAW264.7 murine macrophage cell line, we infected 10^5^ cells per well with a recombinant YS1646 strain expressing mCherry (YS1646::mCherry) at an MOI of 100 and then stained the cells with DAPI. We observed detectable levels of mCherry expression within the macrophages infected with YS1646::mCherry whereas the signal was not detected in our uninfected control (Fig. 1d).

### Heterologous vaccination with nirB_SspH1_CatB leads to robust systemic and local humoral responses

**Fig. 2:**
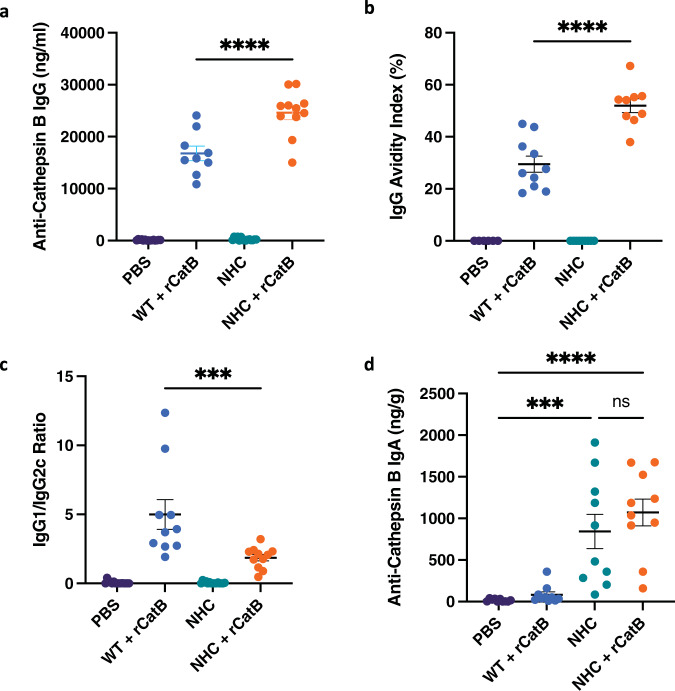
Humoral responses. Serum and intestinal samples were collected 3 weeks post-vaccination. Anti-Cathepsin B IgG titers expressed as ng/mL (**a**). Anti-CatB IgG avidity index (%) (**b**). The ratio of antigen-specific IgG1 vs IgG2c titers (**c**). Anti-CatB intestinal IgA titers expressed as ng/g (**d**). Each independent experiment consisted of five animals per group. Each experiment was performed twice. Data were shown as mean ± SEM. Statistical significance was determined by one-way ANOVA with Tukey’s multiple comparisons (***P < 0.001 and ****P < 0.0001).

We next moved forward with in vivo vaccination studies using our integrated NHC construct in a mouse model. None of the groups had detectable anti-CatB IgG levels at baseline (week 0). Three weeks post-vaccination, no anti-CatB IgG was found in the PBS and PO groups. However, we observed high IgG titers in all mice receiving a single dose of rCatB intramuscularly at 3 weeks. The greatest increase in antigen-specific IgG titers was observed in the multimodal vaccination group, NHC + rCatB, and these antibody levels were significantly higher than in the IM-only group (24,605 ± 1,309 ng/mL vs. 16,783 ± 1,397 ng/mL, *P* < 0.0001) (Fig. 2a). Therefore, multimodal administration led to a greater IgG response to Cathepsin B in vaccinated animals compared to PO or IM dosing alone.

Multimodal vaccination resulted in better IgG avidity maturation as well. At a concentration of 6 M urea, the IgG avidity index (%) was significantly greater in the multimodal group compared to IM vaccination (52.7 ± 2.7% vs. 29.5 ± 3.1%, *P* < 0.0001) (Fig. 2b). The IgG avidity indexes for the PBS and PO groups were not calculated. Next, we measured the titers of anti-CatB IgG subtypes IgG1 and IgG2c. The IgG1/IgG2c ratio was significantly lower in the multimodal group compared to the IM control (1.9 ± 0.2 vs. 5.0 ± 1.1, *P* < 0.001) (Fig. 2c). Since IgG1 titers were comparable between groups, the higher total IgG levels and altered ratio were attributable to an increase in antigen-specific IgG2c levels in mice that received multimodal vaccination.

We then looked at local humoral responses by assessing intestinal CatB-specific IgA titers. All mice that received the NHC-bearing YS1646 orally (with or without rCatB IM) had elevated titers of anti-CatB IgA in the intestinal tissues. Both multimodal vaccination (1,071.0 ± 161.4 ng/g, *P* < 0.0001) and PO vaccination alone (842 ± 206.4 ng/g, *P* < 0.001) led to a significant increase in titers compared to the PBS control group (15.9 ± 5.1 ng/g) (Fig. 2d). There was no statistical difference observed between the NHC alone and multimodal groups. Vaccination with the parental control strain + rCatB led to IgA titers similar to the PBS control (83.1 ± 33.8 ng/g).

We also examined the serum total IgE titers in our study following vaccination. The highest titers were observed in the PBS control group (653.6 ± 83.6 ng/mL) (Supplementary Fig. 2A). Groups that received IM-only and PO-only vaccination had total IgE levels of 387.7 ± 57.0 ng/mL and 444.7 ± 36.6 ng/mL respectively. There was no statistical difference observed between our multimodal vaccination group and any other group in our study. The mean serum total IgE titer in the multimodal group was 457.8 ± 86.1 ng/mL. Thus, none of the vaccination schedules tested increased total IgE levels. Furthermore, no animals developed antigen-specific IgE titers in response to vaccination (Supplemental Fig. 2B).

### Multimodal vaccination induces a mixed T_H_1/T_H_2 cellular response in ex vivo stimulated splenocytes

**Fig. 3:**
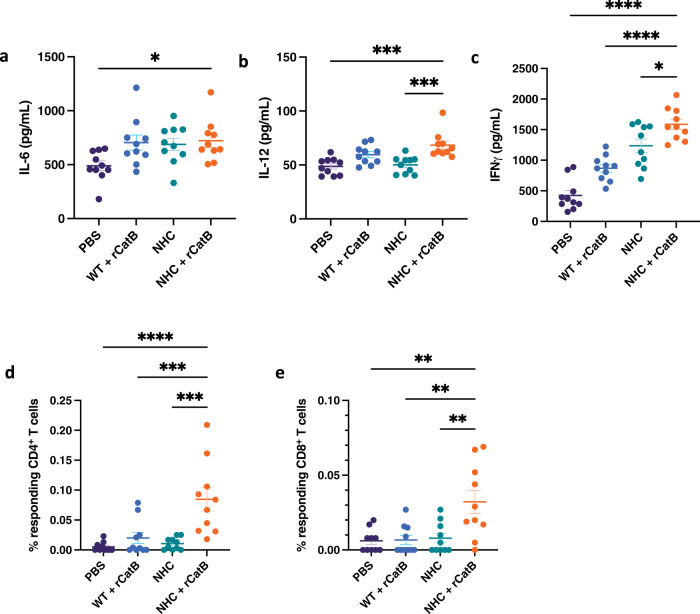
Cellular responses. Supernatant levels of IL-6 (**a**), IL-12 (**b**), and IFNγ (**c**) after ex vivo restimulation of splenocytes with rCatB for 72 h. Cytokines were measured by Quansys multiplex ELISA and expressed in pg/mL. The percentage of responding IFNγ^+^ CD4^+^ T cells (**d**) and IFNγ^+^ CD8^+^ T cells (**e**) in ex vivo restimulated splenocytes with rCatB after 24 h were measured by flow cytometry. Each independent experiment consisted of five animals per group. Each experiment was performed twice. Data were shown as mean ± SEM. For flow cytometric analysis, data are shown as mean ± SEM of net values after background subtraction with unstimulated cells. Statistical significance was determined by one-way ANOVA with Tukey’s multiple comparisons (*P < 0.05, **P < 0.01, ***P < 0.001, and ****P < 0.0001).

Cytokine and chemokine levels in splenocyte supernatants were assessed by Quansys multiplex ELISA following ex vivo restimulation with CatB 3 weeks post-vaccination. There were no significant differences or trends in the levels of IL-1α, IL-1β, IL-2, IL-3, IL-4, TNF-α, CCL3 (MIP-1α), CCL5 (RANTES), or GM-CSF. However, there were significant increases in T_H_1-associated cytokines in the multimodal group compared to the PBS controls. For example, we observed an increase in IL-6 levels (721.3 ± 61.3 pg/mL vs. 489.9 ± 44.3 pg/mL, *P* < 0.05) (Fig. 3a). We also detected increased levels of IL-12 (68.3 ± 3.8 pg/mL) in the multimodal group compared to both the PBS control (48.7 ± 2.5 pg/mL) and PO alone group (50.0 ± 2.5 pg/mL) (both *P* < 0.001) (Fig. 3b). Of note, the strongest CatB-specific IFNγ response occurred in the multimodal group (1,586.6 ± 82.7 pg/mL) compared to the PO group (1,236.2 ± 109.0 pg/mL) (*P* < 0.05), the IM group (869.6 ± 65.2 pg/mL) (*P* < 0.0001), and the PBS control (423.6 ± 79.7 pg/mL) (*P* < 0.0001) (Fig. 3c).

In addition to T_H_1-associated cytokines, we also observed significant differences in the expression of T_H_2, T_H_17, and regulatory cytokines, as well as in chemokine expression with the different vaccination regimens. All mice receiving an IM dose of rCatB had elevated levels of IL-5 that were highest in the multimodal group (230.7 ± 56.5 pg/mL vs. PBS control 12.8 ± 2.9 pg/mL, *P* < 0.01) and only slightly lower in the rCatB IM-only vaccination group (200.1 ± 66.4 pg/mL) (*P* < 0.05 compared to PBS control) (Supplemental Fig. 3A). While not significantly different, there was a trend toward increased IL-10 expression in the NHC + rCatB group (17.2 ± 1.7 pg/mL vs. PBS 11.4 ± 0.8 pg/mL, *P* = 0.096) (Supplemental Fig. 3B). IL-17 secretion levels were also increased in the multimodal vaccination group (116.9 ± 24.7 pg/mL vs. PBS control 41.8 ± 6.6 pg/mL, *P* < 0.01) (Supplemental Fig. 3C). The multimodal vaccine response was also higher than that observed in PO (42.9 ± 4.5 pg/mL, *P* < 0.01) and IM (62.6 ± 8.9 pg/mL, *P* < 0.05) groups. Finally, CCL2 (MCP-1) levels were also higher in the multimodal group compared to the PBS control (2,696.1 ± 107.0 pg/mL vs. 1,859.7 ± 169.9 pg/mL, *P* < 0.01) (Supplemental Fig. 3D).

### Vaccine-induced IFNγ production is mediated by CD4^+^ and CD8^+^ T cells

We next sought to determine whether the increased cytokine levels observed from splenocyte supernatants were generated by T cells. Antigen-specific CD4^+^ and CD8^+^ T cells from vaccinated animals were analyzed by flow cytometry following our multimodal vaccination schedule and restimulation of splenocytes with either rCatB (2.5 μg/mL) or fRPMI medium for 24 h. Responding cells were characterized as CD4^+^ and CD8^+^ T cells expressing IFNγ following our gating strategy (Supplemental Fig. 4).

The percentage of responding IFNγ^+^ CD4^+^ T cells was significantly increased in the multimodal vaccination group (0.08 ± 0.02%) compared to all other groups, including the PBS control (5.0 × 10^−3^ ± 2.5 × 10^−3^%, *P* < 0.0001), the IM group (0.02 ± 9.3 × 10^−3^%, *P* < 0.001), and the PO group (0.01 ± 3.2 × 10^−3^%, *P* < 0.001) (Fig. 3d). Similar but overall slightly lower results were observed in the percentage of responding IFNγ^+^ CD8^+^ T cells: multimodal (0.03 ± 7.8 × 10^−3^%), PBS control (6.1 × 10^−3^ ± 2.4 × 10^−3^%, *P* < 0.01), IM alone (6.7 × 10^−3^ ± 3.1 × 10^−3^%, *P* < 0.01), and PO alone (7.9 × 10^−3^ ± 3.2 × 10^−3^%, *P* < 0.01) (Fig. 3e).

### Multimodal vaccination significantly reduces parasite burden and egg-associated pathology

**Fig. 4:**
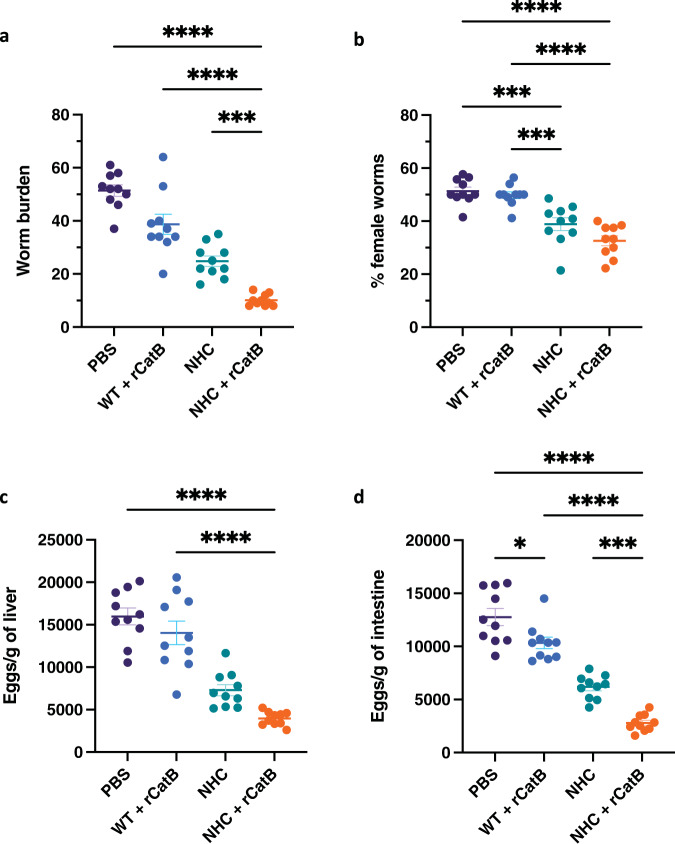
Parasite burden. The worm burden (**a**) and the percentage of female worms (**b**) were assessed 7 weeks post-challenge per individual mouse in each group. Egg loads were expressed per gram of liver (**c**) and intestine (**d**) for each group. Each independent experiment consisted of five animals per group. Each experiment was performed twice. Data were shown as mean ± SEM. Statistical significance was determined by one-way ANOVA with Tukey’s multiple comparisons (*P < 0.05, ***P < 0.001, and ****P < 0.0001).

At 7 weeks post-infection, we assessed the protective potential of our recombinant *Salmonella* vectored vaccine. Mean worm counts were highest in the PBS group (51.4 ± 2.2 worms). Multimodal vaccination significantly reduced worm burden by 80.4% (10.1 ± 0.7 worms, *P* < 0.0001). Compared to the control group, mean worm burdens in the WT + rCatB and NHC groups were reduced by 27.9% (38.7 ± 3.8 worms) and 51.8% (24.8 ± 2.0 worms), respectively. The worm count in the multimodal vaccine group was significantly lower than the WT + rCatB (*P* < 0.0001) and NHC groups (*P* < 0.001) (Fig. 4a). Since female *S. mansoni* worms can lay up to 300 eggs per day^12^, we examined the male-female worm ratio. The percentage of female worms was balanced in the PBS (51.3 ± 1.5%) and the WT + rCatB (49.8 ± 1.3%) groups. In contrast, the proportion of female worms was significantly lower in mice that received oral vaccination with NHC alone (38.8 ± 2.4%, *P* < 0.001 versus PBS) or multimodal vaccination (32.6 ± 1.9%, *P* < 0.0001 versus PBS) (Fig. 4b).

Schistosomiasis pathology is strongly associated with egg deposition in tissue. Hepatic egg burden was highest in the PBS control (15,974 ± 984.7 eggs/g). Multimodal vaccination significantly reduced egg load in the liver by 75.2% (3,965 ± 259.5 eggs/g, *P* < 0.0001). Hepatic eggs were reduced by 17.6% (14,041 ± 1394 eggs/g) in the parental strain control + rCatB group and 54.6% (7292 ± 658.2 eggs/g) in the NHC group (Fig. 4c). Similar observations were made for intestinal egg burden. Compared to the PBS control (12,766 ± 804.3 eggs/g), intestinal egg load in the NHC + rCatB group was reduced by 78.1% (2796 ± 250.5 eggs/g, *P* < 0.0001). Intestinal eggs were reduced by 20.4% (10,331 ± 547.4 eggs/g) in the parental strain control + rCatB group and 51.6% (6185 ± 353.9 eggs/g) in the NHC group (Fig. 4d and Supplemental Table 1).

Histopathological assessment of murine livers showed a larger mean granuloma size (56,536.2 ± 4,727.3 μm^2^) in control mice with a low percentage of abnormal morphology (10.9 ± 1.9%). Mice in the parental strain + rCatB group did not differ greatly from the PBS group in their mean granuloma size (55304.7 ± 4366.9 μm^2^) and percentage of abnormal eggs (15.9 ± 1.4%). Oral vaccination alone with YS1646::NHC significantly reduced the size of granulomas (40,136.1 ± 2,497.6 μm^2^, *P* < 0.05) and mouse livers displayed a higher proportion of abnormal eggs (39.6 ± 2.5%, *P* < 0.0001) compared to the PBS control. The greatest impact was observed in the multimodal vaccination group, which had the significantly smallest egg granuloma sizes (31,620.4 ± 1,759.2 μm^2^, *P* < 0.001) and the highest rate of morphologically abnormal eggs (51.9 ± 4.8%, *P* < 0.0001) (Table 1).

**Table 1:** Granuloma size and egg abnormality.

Vaccine Group	Granuloma size (μm^2^) ± SEM	Abnormal egg morphology (%) ± SEM
PBS	56536.2 ± 4727.3	10.9 ± 1.7
WT + rCatB	55304.7 ± 4366.9	15.9 ± 1.4
NHC	40136.1 ± 2497.6^*^	39.6 ± 2.5^****^
NHC + rCatB	31620.4 ± 1759.2^***^	51.9 ± 4.8^****^

Liver egg granuloma size (μm^2^) and egg morphology (i.e., loss of internal structure, shrinkage, crenellated periphery) were assessed using Zen Blue software. Each independent experiment consisted of 6–10 granulomas per animal per group. Each experiment was performed twice. Data were shown as mean ± SEM.

Statistical significance was determined by one-way ANOVA with Tukey’s multiple comparisons (*P < 0.05, ***P < 0.001, ****P < 0.0001).

## Discussion

Finding the “right” antigen for a schistosomiasis vaccine has been challenging. Indeed, a recent study that screened 96 cell-surface and secreted recombinant *S. mansoni* proteins for efficacy in a murine vaccination-challenge model failed to yield any particularly promising candidates^13^. As noted above, almost all of the candidate vaccines for schistosomiasis developed to date, including those that have entered early clinical development, have targeted surface proteins of adult worms^7^. In contrast, CatB is an important digestive enzyme for *S. mansoni* as it is used to break down host blood macromolecules such as hemoglobin, serum albumin, and IgG. This cysteine protease is expressed in the cecum of migrating schistosomula and in the gut of the adult worm^14^. Suppression of CatB expression by RNA interference leads to significant impacts on parasite growth and survival^15^. Additionally, *S. mansoni* CatB is a highly immunogenic circulating antigen in the sera of infected patients^16^. In previous work, our group has had considerable success targeting CatB with IM formulations of recombinant protein combined with several adjuvants, including CpG dinucleotides^17^, Montanide ISA 720 VG^18^, the MF59-like AddaVax, and sulfated lactosyl archaeal archaeosomes^19^. These studies demonstrated impressive immunological readouts, such as high serum IgG antibody titers and the induction of cellular responses. Parasite burden reductions ranged between 54 to 87%, effectively surpassing the 40% protection threshold proposed in 1998 by the Tropical Diseases Research (TDR) committee of the World Health Organization (WHO)^20^. In parallel with this work, we have developed an alternate strategy to deliver Cathepsin B to the host by PO vaccination with an attenuated *Salmonella* Typhimurium strain alone or by multimodal vaccination. Unlike traditional IM vaccination approaches, we hoped this approach would elicit both mucosal and systemic responses. Using a prime-boost strategy with an oral YS1646-vectored dose followed 30 days later by an IM dose, reductions of liver and intestinal parasite burden as high as 93% were observed following challenge^8^. We subsequently established the therapeutic efficacy of multimodal YS1646 vaccination using a shortened, 5-day regimen (PO + IM vaccination on Day 1 followed by 2 PO doses on Days 3 and 5), with efficacy rates of up to 73% in a chronic schistosomiasis infection model^9^. This strategy, therefore, meets the more recent criteria established by global experts for a desirable schistosomiasis vaccine (i.e., 75% efficacy when used prophylactically with an additional therapeutic effect)^21^. Using the same shortened multimodal vaccination schedule, the YS1646::NHC strain with a chromosomally integrated expression system generated in our present study also meets these criteria, with an 80% reduction in worm burden and a 75/78% reduction in hepatic/intestinal egg burden.

While suitable for proof-of-concept murine studies^8,9^, vaccination using plasmid-based *Salmonella* YS1646 would not be suitable for human use. This is due to the risk of spreading antibiotic resistance as well as unintended effects on the microbiota of the host^22,23^. In the field of DNA vaccines, plasmids for live bacterial vectors are recommended to be marker-free^24^. Additionally, with a mid-range copy number^25^, the plasmid may be lost during cell division, especially in non-selective conditions^26,27^. Chromosomal integration of our gene construct into the genome of *S*. Typhimurium also allows for more stable and reliable expression^11^. Through Tn7 transposition, we were able to integrate the vaccine antigen-encoding gene fusion at the *att*Tn7 site, which is downstream of the *glmS* gene, which encodes an essential glucosamine-fructose-6-phosphate aminotransferase^28^. This gene plays an important role in *Salmonella* cell envelope integrity^29^. In addition, single-copy integration of our constructs was also effective as it did not affect bacterial viability and resulted in strong humoral and cellular immune responses. As *Salmonella* Typhimurium targets host macrophages as part of its life cycle^30^, infection of RAW264.7 macrophage cells with mCherry-expressing YS1646 also served as an indicator for the likelihood of CatB expression from the promoters selected for use when the *S*. Typhimurium YS1646 vector strain was located within macrophages.

The use of a *Salmonella* delivery platform is attractive for several reasons. The natural presence of bacterial LPS and flagellin proteins can serve to stimulate the immune system and act as “auto-adjuvants” through binding to TLR-4 and TLR-5, respectively^31–33^. The YS1646 strain has previously been used in phase I clinical trial for the treatment of metastatic melanoma and was well tolerated in patients at doses up to 3 × 10^8^ CFU intravenously^34^. Live attenuated *Salmonella* vectored vaccines have been designed against other infectious diseases, including a commercial typhoid vaccine (Ty21a)^35^ and several preclinical candidates for other parasitic infections^36–38^. *Salmonella enterica* uses its type 3 secretion system (T3SS) to invade specialized gut epithelial cells^39^. Host cell entry is mediated by early effector proteins secreted by the *Salmonella* pathogenicity island I (SPI-I) T3SS, whereas the SPI-II T3SS is active to secrete late effector proteins once the bacterium is within its *Salmonella*-containing vacuole (SCV)^40^. The SspH1 secretion signal can mediate the secretion of recombinant target antigens through both SPI-I and SPI-II T3SSs. Overall, the use of live attenuated *Salmonella* as vaccine vectors is a proven strategy. In fact, the *S*. Typhi commercial vaccine (Vivotif) is administered as a set of four oral doses within the span of a week, which is very similar to the oral component of our multimodal vaccination strategy.

Developing the “ideal” schistosomiasis vaccine in the context of desired immune responses is complex. While much work has been done to study the immunopathogenesis of the disease, precise correlates of immunity remain to be elucidated. However, a large body of preclinical data has helped determine possible characteristics of an effective vaccine. Rhesus macaques represent a unique animal model due to their ability to self-cure at the onset of egg deposition by mature worms^41^. There has been renewed interest in studying this model to determine what constitutes a protective immune profile. From these studies, it has been postulated that high antibody titers against key target antigens create an environment of sustained immune pressure which results in reduced rates of worm survival^42^. This immunological pressure may lead to physiological damage of the adult worms^43^ and may induce the autophagic machinery of the parasite as has been observed under other stress conditions such as starvation or drug treatment^44–46^. In fact, Sm-Cathepsin B IgG antibodies target the parasite’s ability to digest host blood macromolecules. It is plausible that the high IgG titers observed in our study lead to decreased worm fitness, which correlates with the changes in egg morphology observed in our prior work^8,9,47^. Indeed, the induction of high titers of anti-CatB IgG by adjuvanted IM vaccination has been previously demonstrated and may correlate with reduced parasite burden^48^. The supportive role of the humoral response has also been observed in other mouse models^49^. The potential neutralizing effect on Sm-Cathepsin B of vaccine-elicited antibody responses remains to be elucidated. Interestingly, our multimodal vaccination approach with the YS1646-based single-copy recombinant vaccine elicited stronger IgG titers than previously observed in our plasmid-based system despite what is likely to be far fewer copies of the CatB gene/bacterium^8^. We also measured higher IgG avidity maturation in the NHC + rCatB group, suggesting there may be important differences in the antibodies generated by multimodal vaccination compared to the IM control. Furthermore, multimodal vaccination led to a more balanced IgG subtype profile with higher titers of antigen-specific IgG2c titers. Recombinant CatB tended to elicit a T_H_2-skewed response, while the *Salmonella* vector caused a T_H_1 bias, leading to a more “balanced” humoral response.

Oral administration of our recombinant YS1646-based vaccine generated a strong local, mucosal response in the gastrointestinal tract. Although schistosomes are not generally considered to be mucosal pathogens, migrating schistosomula transit through the respiratory mucosa of the lungs and adult worms reside in the mesenteric venules of the small intestine. Both parasite life cycle stages interact with mucosal-associated lymphoid tissues (MALT) and the protective potential of IgA antibodies has previously been reported for schistosomiasis^50,51^. Indeed, lung-stage schistosomula represent one of the most vulnerable stages in the life cycle and are subject to attack by eosinophils^52–54^. Secreted IgA antibodies may work in concert with eosinophils in schistosomula killing by mediating multiple effector functions^55^, and eosinophils have also been shown to contribute to the maintenance of IgA^+^ plasma cells^56^. While systemic immune responses are crucial to protect against schistosomiasis, the induction of local, mucosal responses may be beneficial and represent a currently underutilized strategy. Allergic-like type I hypersensitivity responses have been detrimental in other helminth vaccination studies^57^. As observed in our therapeutic vaccination model^9^, total IgE levels were comparable across all groups and multimodal vaccination did not significantly increase IgE titers. The lack of antigen-specific IgE is also a promising feature. Nonetheless, the study of IgE antibody responses to CatB would require further evaluation in a clinical setting to better assess the impact on humans.

T_H_1 cellular responses have been established as a key feature of schistosomiasis vaccine development in murine models^17,58,59^. However, both exaggerated T_H_1- and T_H_2-biased responses can lead to severe pathology and death^1^. Thus, the optimal T cell immunity would likely be a balanced and targeted T_H_1/T_H_2 response. Upon ex vivo restimulation of splenocytes from vaccinated animals with rCatB, we measured high levels of several T_H_1-type cytokines in supernatants such as IL-6, IL-12, and IFNγ. These T_H_1 immune responses are likely due to the influence of the YS1646 vector. IFNγ is generally regarded as a hallmark of schistosomiasis protection based on findings using radiation-attenuated schistosome vaccines^60,61^. Levels of IFNγ were significantly increased in the multimodal group compared to all other groups in our study. The increased production of IFNγ in mice receiving multimodal vaccination is likely due to production by both CD4^+^ and CD8^+^ T cells, as demonstrated by our flow cytometry experiments. IL-6 and IL-12 may serve to promote a more pro-inflammatory environment. Increases in IL-12 secretion, although small, are quite significant due to the tight control exerted on this regulatory cytokine by the immune system^62^. IL-12 has even been considered as a potential adjuvant in schistosomiasis vaccine studies^63^. In addition, we also observed significant increases in IL-5, IL-17, and CCL2, along with a trend toward an increase for the regulatory cytokine IL-10. IL-5 is implicated in the recruitment of eosinophils as well as playing a role in IgA secretion by plasma cells^64–66^. Together with IL-10, IL-5 may serve to dampen the pro-inflammatory environment induced by T_H_1-type cytokines and generate a more balanced cellular response. The T_H_17 pathway has been associated with lower worm burdens and IL-17 secretion has been correlated with the recruitment of neutrophils which have an impact on migrating schistosomula via extracellular traps^67^. T_H_17 cytokine expression in peripheral blood mononuclear cells (PBMCs) has also been demonstrated in a baboon animal model testing an Sm-p80 vaccine^68^. CCL2 is a chemokine responsible for the recruitment of monocytes, memory T cells, and dendritic cells, which may serve to further promote a T_H_1-T_H_2 environment^69,70^.

Parasite burden reduction rates achieved by the chromosomally integrated antigen expression and delivery system in strain YS1646::NHC were comparable with our previous plasmid-based prime-boost vaccination strategy^8^ despite a much shorter schedule. These candidate vaccines meet the recently developed Preferred Product Characteristics (PPC) of a schistosomiasis vaccine by reaching the 75% target for worm and egg burden reductions^21,71^. Our results surpass those of other vaccines in comparable murine models that have since entered clinical trials^72^. Recombinant Sm-TSP2 achieved a 57% reduction in adult worms and a 64% reduction in hepatic eggs, whereas recombinant Sm14 reduced worm burden by 66%, both adjuvanted with Freund’s complete adjuvant^73,74^. Recently, recombinant Sm-p80 adjuvanted with GLA-SE achieved a 93% reduction in female worms and 90% in tissue egg load in a non-human primate model^72^. Mouse studies of this vaccine demonstrated a 70% reduction in parasite burden^71^. These vaccine strategies require repeated intramuscular immunizations, whereas the schedule used in the present study required only 5 days. A striking result of the chromosomally integrated antigen expression-based vaccine was the significant reduction in the percentage of female worms in the multimodal group, suggesting that this approach may have an anti-fecundity effect. This finding is consistent with the reduced egg granuloma sizes and the higher proportion of morphologically abnormal eggs observed as well as in previous Cathepsin B vaccination studies^8,9,19,47^. Therefore, we hypothesize that adult female worms are under higher immune and metabolic pressure in vaccinated animals and this can lead to decreased fitness and egg integrity.

The focus of this work was the characterization of the protective potential and immune responses generated by a single-copy chromosomally integrated antigen expression-based attenuated Salmonella strain as an *S. mansoni* vaccine candidate. While the current work represents considerable progress in the development of an *S. mansoni* vaccine more suitable for human use (i.e., more stable antigen expression & removal of antibiotic resistance), several possible limitations of this approach remain. The stability of our YS1646::NHC strain for long-term storage remains to be evaluated and it is not yet known if a cold chain would be required. To address this concern, we freeze-dried our vaccine following a similar procedure used for the attenuated S. typhi vaccines, Ty21a^75^, and measured CFU concentrations over the course of 16 weeks to assess room temperature (RT) stability (Supplemental Fig. 5). Despite an initial drop of 1 log following lyophilization, concentrations remained stable for 8 weeks until a small decrease of a half log at 12 weeks, which was then maintained at 16 weeks. Additional experiments will be required to measure antigen expression and retention of immunogenicity in freeze-dried preparations at different time points. Furthermore, previous work using rCatB has highlighted the benefit of adjuvants, while our studies with YS1646 to date have included none. The in-built adjuvanticity of the *Salmonella* vector via its TLR agonizts likely helped induce an immune response against CatB, however, future studies may include an adjuvant for either PO or IM doses to further enhance immunogenicity and protection. Given the local mucosal response conferred by PO doses and protection against challenges previously observed with oral immunization alone, we hope to explore the possibility of a YS1646-derived vaccine without intramuscular rCatB doses, creating a needle-free vaccine. Future studies will include mechanistic experiments to elucidate correlates of immunity related to our vaccine. Lastly, *S*. Typhimurium can disseminate widely in the murine host and persist for weeks^76^. Phase I clinical studies with YS1646 demonstrated that the bacterium could not survive long in the host’s bloodstream due to its susceptibility to physiologic levels of CO_2_^77^. As we consider clinical studies, we hope that short-lived local invasion of the gastrointestinal tract will help induce potent immune responses while limiting persistence and dissemination within vaccinated individuals. Finally, there is 60% homology between schistosomal and mammalian Cathepsin B, which raises concerns surrounding off-target effects. However, the mammalian homolog is restricted to the cellular lysosomal compartment^78^, rendering cross-reactivity of vaccine-induced antibodies less likely.

In summary, we report the development of a chromosomally integrated multimodal YS1646-vectored vaccine expressing *S. mansoni* Cathepsin B that can induce a superior immune response in a mouse model compared to previous plasmid-based formulations with high parasite burden reductions. Oral dosing with *S*. Typhimurium expressing a single copy of our target antigen in combination with a single, intramuscular, unadjuvanted dose of recombinant protein led to strong local and systemic immune responses. The protection achieved against several parasitological outcomes ranges among the best reported in the murine model. As we look toward clinical studies and implementation of the vaccine, we expect that administration of our multimodal vaccine will be easier in clinical practice compared to other schistosomiasis vaccination strategies that rely on several intramuscular doses delivered over a period of many months. A single doctor’s visit would include the administration of the IM dose along with the first PO dose. Then, patients could continue their oral vaccine regimen at home, similar to the Ty21a *S*. Typhi vaccine, which consists of four oral doses^35^. This would greatly facilitate vaccination of those living in endemic regions for schistosomiasis where recurrent visits to the clinic may be challenging^79,80^. Chromosomal integration of CatB in the YS1646 vector also allowed the removal of the plasmid-located antibiotic resistance gene, making our candidate vaccine much safer for eventual clinical use. These data strongly support the continued development of this candidate *S. mansoni* vaccine to potentially aid the hundreds of millions of people presently infected or at risk worldwide.

## Methods

### Ethics statement

All animal procedures were conducted in accordance with Institutional Animal Care and Use Guidelines and were approved by the Animal Care and Use Committee at McGill University (Animal Use Protocol 7625) as well as the Canadian Council on Animal Care.

### Bacterial plasmids

*Salmonella enterica* Typhimurium (*S*. Typhimurium) strain YS1646 (Δ*msbB2* Δ*purI* Δ*Suwwan xyl* negative; ATCC 202165; ATCC, Manassas, VA) was obtained from Cedarlane Labs (Burlington, ON, Canada). Recombinant Tn7 plasmids were produced in *Escherichia coli* DH5α π kindly provided by Dr. Charles Dozois. Conjugation experiments with YS1646 required *E. coli* MGN-617 for donor genetic material^81^. Plasmids were introduced into YS1646 either by conjugation or by electroporation (20 ng of plasmid at 1.8 kV, 200 Ω, and 25 μF; ECM 399 Electroporation System, BTX, Holliston, MA, USA). Plasmids were introduced into *E. coli* by heat shock. *S*. Typhimurium and *E. coli* were cultured in Luria broth (LB), with the following antibiotics and amino acids when necessary to maintain plasmids: 100 μg/mL ampicillin (Amp), 50 μg/mL kanamycin (Km), 30 μg/mL chloramphenicol (Cm), and 50 μg/mL diaminopimelic acid (DAP).

### Chromosomal integration

The *nirB* and *pagC* promoters, as well as the SspH1 and Cathepsin B (codon-optimized for expression in *S*. Typhimurium) sequences, were utilized in this study^8,82^. The *frr* promoter was obtained from YS1646 *S*. Typhimurium by PCR. The pGP-Tn7-Cm plasmid backbone^11^ was digested using FastDigest restriction enzymes EcoRI and KpnI (Thermo Fisher Scientific). The promoter, secretory signal, and antigen sequences were inserted using the *pEASY*—Uni Seamless Cloning and Assembly kit (TransGen Biotech, Beijing, China). Following the construction of novel Tn7 plasmids, DAP^−^ *E. coli* MGN-617 was transformed to generate 3 novel conjugative donor strains. The transformed *E. coli* strains were then used for conjugation experiments with a YS1646 strain containing the temperature-sensitive pSTNSK plasmid which encodes the Tn7 transposase system and confers Km resistance^11^. Donor *E. coli* and recipient YS1646 were resuspended in LB supplemented with Km and DAP. About 100 μL of the donor strain and 50 μL of the recipient strain were centrifuged together and then resuspended in 10 μL at 30 °C for 5 h. Mixed culture was later grown on LB Km-Cm plates at 37 °C, and YS1646 colonies that grew in the absence of DAP, but that had lost resistance to antibiotic markers present on the pSTNSK plasmid but gained Cm resistance associated with the *att*Tn7 targeting sequence were selected. Chromosomal integration at the *att*Tn7 was then confirmed by PCR. The temperature-sensitive pCP20 plasmid, encoding the recombinase flippase (FLP) and conferring Amp and Cm resistance, was transformed into YS1646 strains. The Cm resistance cassette, integrated at the *att*Tn7 site, was flanked by two FRT regions. Loss of the Cm resistance cassette is mediated by FLP-FRT recombination. Transformants were selected on LB-Amp plates at 30 °C to maintain pCP20 activity and then serially passaged on LB-Amp at 30 °C as well as LB-Cm and LB at 37 °C to screen for loss of the pCP20 plasmid and antibiotic susceptibility. Loss of Cm resistance was further confirmed by PCR. Following a similar strategy as outlined above, mCherry-expressing strains were generated for confocal microscopy experiments.

### Growth curves

Cultures of wild-type YS1646 and chromosomally integrated constructs were grown overnight at 37 °C. The next day, the cultures were diluted 1:100 in LB medium and plated in quadruplicates (*n* = 4) on a 100-well Bioscreen C honeycomb microplate (Growth Curves USA, Piscataway, NJ, USA). The Bioscreen C plate reader measured the optical density of the cultures at a wavelength of 600 nm every 30 min over 24 h with a 30-s shaking period prior to each reading.

### Lyophilization of *Salmonella* strains

YS1646-derived vaccine candidates and control strains were first cultured in LB medium and then formulated in a lyophilization buffer consisting of 28% (w/v) sucrose (BioShop, Burlington, Canada) and 1% gelatin (Sigma-Aldrich, St. Louis, MO) in 25 mM potassium phosphate (KPO_4_) at pH 7^75^. Samples were aliquoted and frozen at −80 °C overnight. The following day, the strains were freeze-dried using a ModulyoD Freeze Dryer (Thermo Fisher Scientific) for 24 h. Samples were later stored at room temperature for various times ranging from 2 weeks to 4 months and cultured on LB plates to determine viability through the colony-forming unit (cfu) counts. A sample that did not undergo the freeze-drying process was cultured to determine the initial viability of strains (determined by cfu counts) prior to lyophilization.

### Confocal microscopy

Murine macrophage-like cells (RAW264.7; ATCC TIB-71) were propagated and cultured for infection with YS1646 strains^8^. Briefly, cells were seeded in an eight-well chamber slide at a concentration of 10^5^ cells/well in Dulbecco’s Modified Eagle medium (DMEM) (Wisent Bioproducts, St-Bruno, QC) supplemented with 10% FBS (Wisent Bioproducts). Recombinant YS1646 strains were diluted in DMEM-FBS to achieve a multiplicity of infection (MOI) of 50 or 100. After 1 h of infection at 37 °C and 5% CO_2_, wells were washed with PBS (Wisent Bioproducts) and incubated with DMEM-FBS supplemented with 50 μg/mL gentamicin (Sigma-Aldrich) for 2 h. The wells were then washed again with PBS and the gentamicin concentration was lowered to 5 μg/mL for overnight incubation. Following this, infected cells were fixed with 2% paraformaldehyde in PBS for 10 min at room temperature. Cells were washed with PBS and then stained with a 1:1000 dilution of 4′,6-diamidino-2-phenylindole (DAPI) (Thermo Fisher Scientific) for an additional 10 min at RT. Images were obtained using a Zeiss LSM780 laser scanning confocal microscope and analyzed using ZEN blue software (Zeiss, Oberkochen, Germany).

### Mouse immunization

Female 6–8-week-old C57BL/6 mice were immunized orally (PO) with recombinant *S. enterica* Typhimurium YS1646 derivative wherein the chromosomally integrated (CI) *nirB* promoter was used for expression and *S. mansoni* Cathepsin B that was secreted by fusion of CatB to an SspH1 *Salmonella*-specific type three secretory signal sequence (YS1646::NHC). This construct was selected for further in vivo investigation based on preliminary animal studies. PO dosing, consisting of 200 μL containing 1 × 10^9^ cfu/dose, was administered by oral gavage every other day for 3 days (D1, D3, and D5). The multimodal vaccination schedule also included a simultaneous intramuscular (IM) dose on D1 of 20 μg recombinant CatB (rCatB) in 50 μL PBS^9^ (Supplemental Fig. 1). Briefly, purified recombinant *S. mansoni* CatB was cloned and expressed in *Pichia pastoris*^17^. All experiments included a PBS control group with IM-only (wild-type (WT) + rCatB) and PO-only (YS1646::NHC) groups as additional controls. The number of animals used at each experimental endpoint is indicated in the Figure legends.

### Evaluation of humoral responses by enzyme-linked immunosorbent assay (ELISA)

Cathepsin B-specific IgG was assessed by ELISA^8^. Serum was collected by saphenous vein bleed at baseline (week 0) and 3 weeks post-vaccination in microtainer serum separator tubes (BD Biosciences, Mississauga, ON, Canada). Serum samples were obtained following the manufacturer’s instructions and stored at −20 °C until assayed. U-bottom, high-binding 96-well plates (Greiner Bio-One, Frickenhausen, Germany) were coated overnight at 4 °C with 0.5 μg/mL rCatB in a 100 mM bicarbonate/carbonate buffer at pH 9.6. Purified mouse IgG (Sigma-Aldrich) was used to set a 2-fold standard curve at a starting concentration of 2000 ng/mL. All samples were run in duplicate. Serum samples were diluted 1:50 (IgG) or 1:20 (IgE) and HRP-conjugated anti-mouse IgG (Cat. No. 12-349; Sigma-Aldrich) was diluted 1:20,000 in blocking buffer (PBS + 2% BSA). HRP-conjugated anti-mouse IgE (Cat. No. PA1-84764; Thermo Fisher Scientific) was diluted 1:6000 in the blocking buffer. Optical density (OD) was measured at 450 nm with an EL800 microplate reader (BioTek Instruments Inc., Winooski, VT). The concentrations of CatB-specific total IgG were calculated by extrapolation from the standard curve. These results are expressed as ng/mL. CatB-specific IgE results are expressed as OD_450_ values.

Cathepsin B-specific IgG avidity was assessed following a modified version of the IgG ELISA protocol described above. Following the primary antibody incubation step, plates were washed 4x with phosphate-buffered saline (PBS) and a range of urea concentrations (0–10 M) was applied to each plate for a 15-min incubation at room temperature (RT). After washing plates 4x, they were incubated for 1 h in a blocking buffer. The standard IgG ELISA protocol then resumed with the addition of the secondary antibody. The IgG avidity index was calculated by dividing urea-treated bound IgG titers with IgG titers assessed in the absence of urea.

CatB-specific IgG subtypes, IgG1 and IgG2c, were assessed by ELISA^9^. Purified mouse IgG1 (Sigma-Aldrich) and a mouse IgG2c isotype control (Southern Biotechnologies Associates, Birmingham, AL) were used to set the respective standard curves. ELISA protocol was run as above for total IgG. Either goat anti-mouse IgG1 or goat anti-mouse Ig2c, both diluted 1:20,000, were used as secondary antibodies (Cat. No. 1070-05 (IgG1); Cat. No. 1079-05 (IgG2c); Southern Biotechnologies Associates). These results are presented as the ratio of IgG1/IgG2c.

Intestines were collected three weeks post-vaccination and processed^8^. Purified mouse IgA (Sigma-Aldrich) was used to set a two-fold standard curve starting at a concentration of 1000 ng/mL. Tissue samples were previously diluted 1:5 (w/v) during the processing phase. Aliquots were applied neat to the plate and the anti-mouse IgA secondary antibody (Cat. No. 62-6720; Thermo Fisher Scientific) was diluted 1:2000. The concentrations of CatB-specific total IgG were calculated by extrapolation from the standard curve. These results are expressed as ng/gram of intestinal tissue.

The BD OptEIA™ Mouse IgE ELISA Set (BD, San Diego, CA) was used to determine total serum IgE titers following the manufacturer’s instructions^9^. Immulon 2HB flat-bottom 96-well plates (Thermo Fisher Scientific) were coated with anti-mouse IgE capture antibody at a 1:250 dilution in coating buffer at 4 °C overnight. Standard concentrations were added following the manufacturer’s protocol. Serum samples were diluted 1:20. OD_450_ was measured and the concentration of total IgE titers was calculated by extrapolation from the standard curve. These results are expressed as ng/mL.

### Cell-mediated responses

CatB-specific cytokine and chemokine production were assessed 3 weeks post-vaccination in splenocytes restimulated ex vivo with 2.5 μg/mL of rCatB for 72 h at 37 °C and 5% CO_2_^9^. Cells were plated 10^6^/well in RPMI-1640 (Wisent Bioproducts) supplemented with 10% fetal bovine serum, 1 mM penicillin/streptomycin, 10 mM HEPES, 1X MEM non-essential amino acids, 1 mM sodium pyruvate, 1 mM l-glutamine (all from Wisent Bioproducts), 0.05 mM 2-mercaptoethanol (Sigma-Aldrich) (fancy RPMI, fRPMI). Supernatant cytokine/chemokine levels were measured using the Q-plex Mouse Cytokine-Screen (16-plex) multiplex ELISA following the manufacturer’s recommendations (Quansys Biosciences, Logan, UT). This assay included: IL-1α, IL-1β, IL-2, IL-3, IL-4, IL-5, IL-6, IL-10, IL-12p70, IL-17, IFNγ, TNFα, MCP-1 (CCL2), MIP-1α (CCL3), GM-CSF, and RANTES (CCL5).

CatB-specific CD4^+^ and CD8^+^ T cells were identified from freshly isolated splenocytes plated 10^6^/well in 96-well round bottom plates (Fisher Scientific) and incubated for 24 h at 37 °C and 5% CO_2_ for flow cytometry analysis^83^. For the last 5 h of incubation, Golgi Stop and Golgi Plug (BD Biosciences) were added to samples according to the manufacturer’s instructions to inhibit protein transport. Cells treated with phorbol 12-myristate 13-acetate (PMA) and ionomycin were included as positive controls. All centrifugation steps occurred at 400×*g* for 7 min at 4 °C until the cells were fixed after which rotor speed was increased to 450×*g*. All incubation steps required light protection. Cells were washed twice with PBS and later stained with Fixable Viability Dye eFluor 780 (eBioscience, Waltham, MA) for 15 min at 4 °C. Cells were washed 2× and incubated with Fc Block (BD Biosciences) diluted 1:50 in PBS for 10 min at 4 °C. Cells were then stained for 25 min at 4 °C with surface markers from the following extracellular cocktail: anti-CD3 FITC (1 μL/sample, eBioscience Cat. No. 11-0031-86), anti-CD4 V500 (1 μL/sample, BD Biosciences Cat. No. 560782) anti-CD8 PerCP-Cy5.5 (1μL/sample, BD Biosciences Cat. No. 551162), anti-CD44 BUV395 (1 μL/sample, BD Biosciences Cat. No. 740215) anti-CD62L BUV373 (0.5 μL/sample, BD Biosciences Cat. No. 612833). Cells were then washed with PBS and fixed with 1X Fixation Buffer (BD Biosciences) overnight at 4 °C. The next day, cells were washed with perm/wash buffer (BD Biosciences) and stained with anti-IFNγ PE (1 μL/sample, BD Biosciences Cat. No. 562020) for 25 min at 4 °C. Cells were washed in perm/wash buffer, then PBS, and later resuspended in PBS for acquisition. All flow cytometry was conducted using a BD LSRFortessa X20 cell analyzer. Data were analyzed using FlowJo software (Treestar, Ashland, OR).

### *Schistosoma mansoni* challenge

*Biomphalaria glabrata* (M-line) infected with the *S. mansoni* Puerto Rican strain (PR-1) were obtained from the Schistosomiasis Resource Center of the Biomedical Research Institute (Rockville, MD) through NIH-NIAID Contract HHSN272201700014I for distribution through BEI Resources. Mice were challenged 3 weeks following vaccination with 150 cercariae by tail exposure and were sacrificed 7 weeks post-challenge^84^. Briefly, adult worms were counted after perfusion of the hepatic portal system and manual removal from the mesenteric veins. The livers and intestines were harvested from each mouse, weighed, and digested in 4% potassium hydroxide overnight at 37 °C. The next day, the number of eggs per gram of tissue was recorded by microscopy.

### Histopathological assessment by H&E staining

Liver sections were harvested and placed in 10% phosphate-buffered formalin (Fisher Scientific) and processed for histopathology to assess mean egg granuloma size and morphology using Zen Blue Software (version 2.5.75.0; Zeiss)^8^. Mean areas are presented as ×10^3^ μm^2^ ± standard error of the mean (SEM). Egg morphology was classified by an operator blinded to group assignment (ASH) as abnormal if there was a loss of internal structure or peripheral crenelation. Abnormal eggs are reported as a percent of the total egg count (±SEM) per histopathological sample.

### Statistical analysis

Statistical analysis was performed using GraphPad Prism 9 software (La Jolla, CA). Results are represented by at least two independent experiments (see Figure legends for details). Data were analyzed by one-way ANOVA and followed by Tukey’s correction for multiple comparisons. *P* values less than 0.05 were considered significant.

### Reporting summary

Further information on research design is available in the Nature Research Reporting Summary linked to this article.

## Supplementary information

Supplemental Material

REPORTING SUMMARY

## Data Availability

Data generated in the current study are available from the corresponding author.
