# Supplementary material for: Salmonella Typhimurium expressing chromosomally integrated Schistosoma mansoni Cathepsin B protects against schistosomiasis in mice
Source: NPJ Vaccines. 2023 Feb 27;8:27. doi: 10.1038/s41541-023-00599-w (PMC9969381; doi:10.1038/s41541-023-00599-w)
Supplement: Supplementary file 1 — Supplemental Material [file 41541_2023_599_MOESM1_ESM.pdf]

Supplemental material

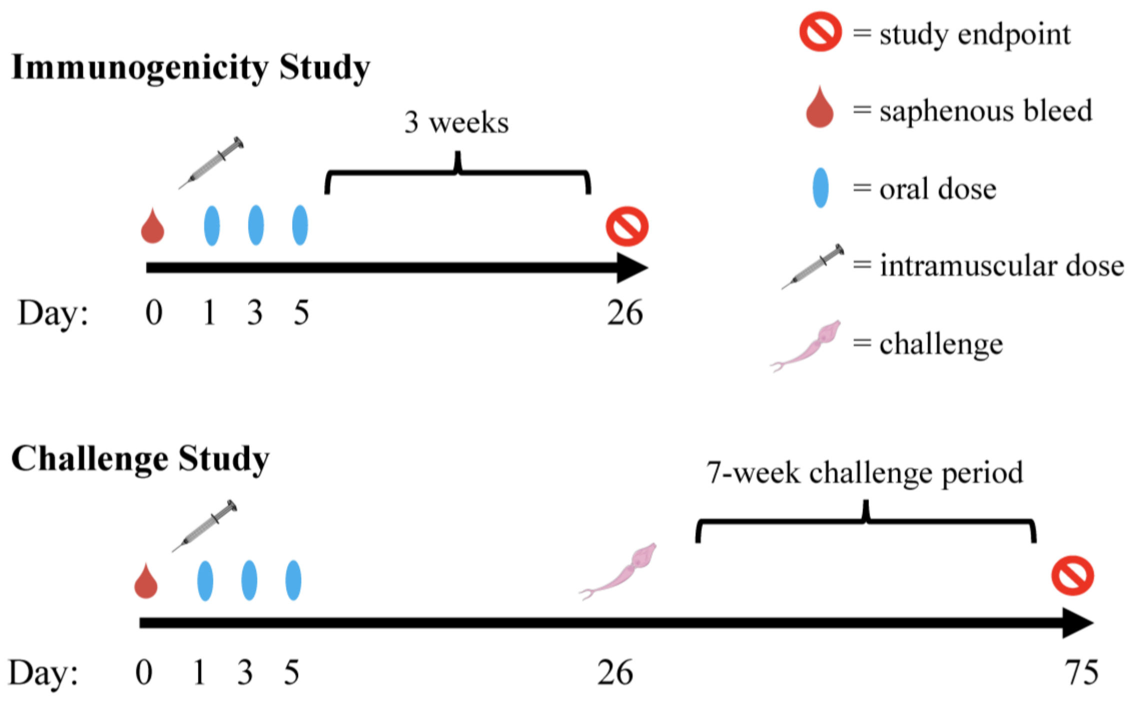

Vaccine Group	Oral Dose	Intramuscular Dose
PBS	PBS	PBS
WT + rCatB	WT YS1646	rCatB
NHC	YS1646::NHC	PBS
NHC + rCatB	YS1646::NHC	rCatB

Supplemental Figure 1. Study Design. Baseline serum was collected on D0 for all mice. Depending on the experimental group, mice received three oral doses of PBS, WT YS1646 or YS1646::NHC on D1, D3, and D5 (200 µL, 1 x 10^9 CFU/dose). Mice also received an intramuscular dose of PBS or rCatB (50 µL, 20 µg/dose). Mice were then euthanized three weeks post-vaccination for immunogenicity studies. For challenge studies, mice were percutaneously exposed to 150 cercaria and euthanized 7 weeks later to assess parasite burden.

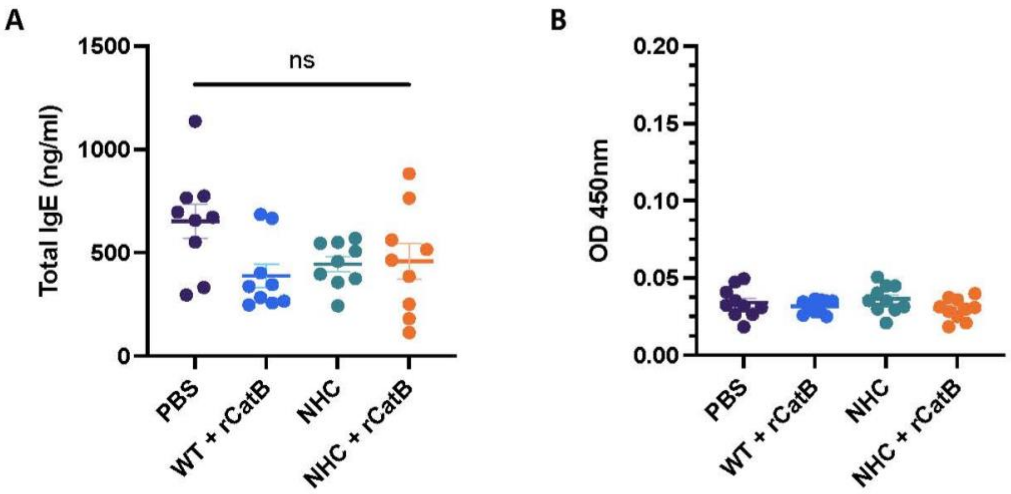

Supplemental Figure 2. Total and antigen-specific IgE. Serum samples were collected 3 weeks post-vaccination. Total serum IgE (A) and antigen-specific IgE (B) in response to vaccination. Each independent experiment consisted of 5 animals per group. Each experiment was performed twice.

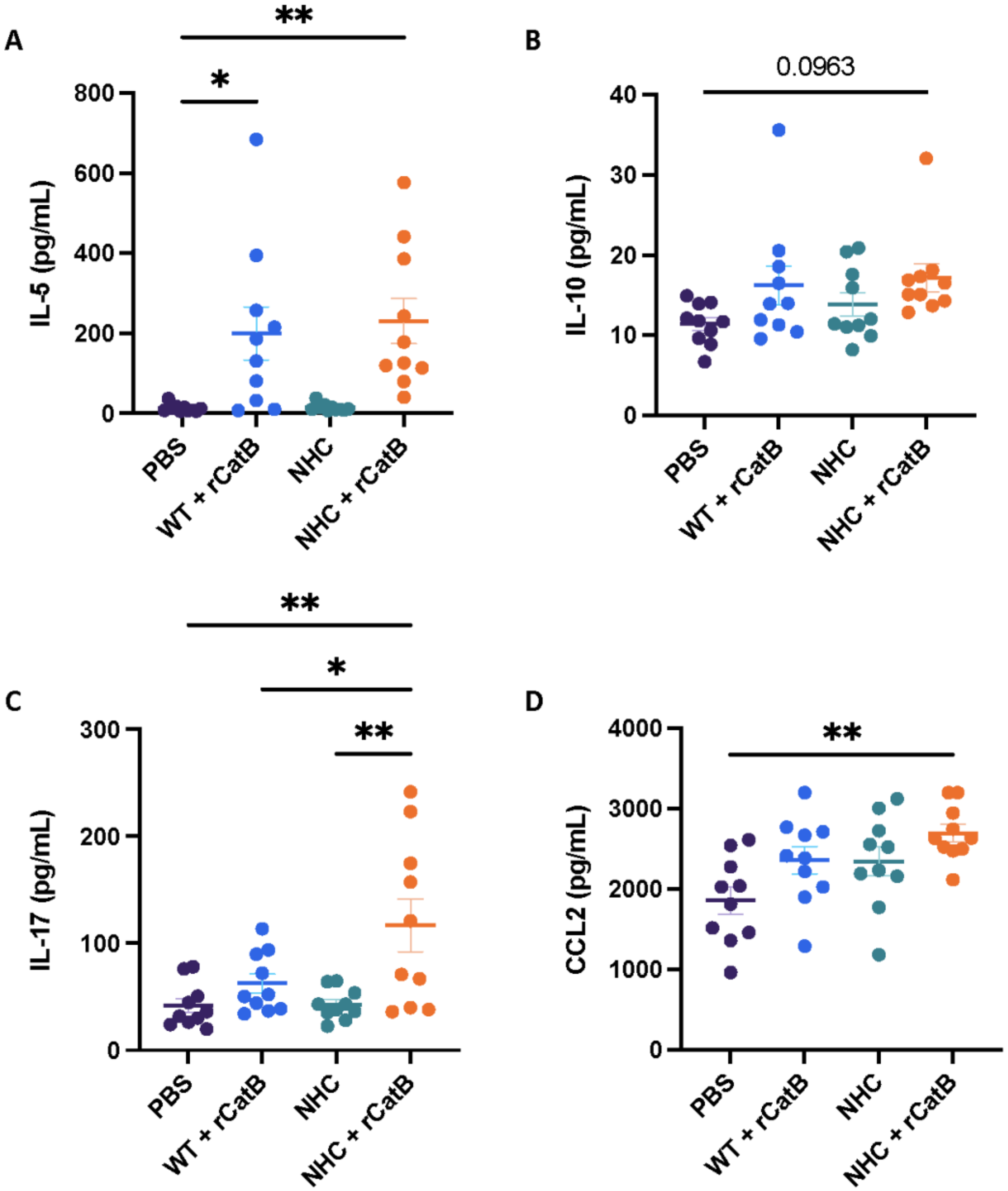

Supplemental Figure 3. Other cytokines and chemokines. Supernatant levels of IL-5 (A), IL-10 (B), IL-17 (C), and CCL2 (D) after *ex vivo* restimulation of splenocytes with rCatB for 72 hours. Cytokines were measured by Quansys multiplex ELISA and expressed in pg/mL. Each independent experiment consisted of 5 animals per group. Each experiment was performed twice. Data are shown as mean ± SEM. Statistical significance was determined by one-way ANOVA with Tukey's multiple comparisons (*$P < 0.05$, **$P < 0.01$).

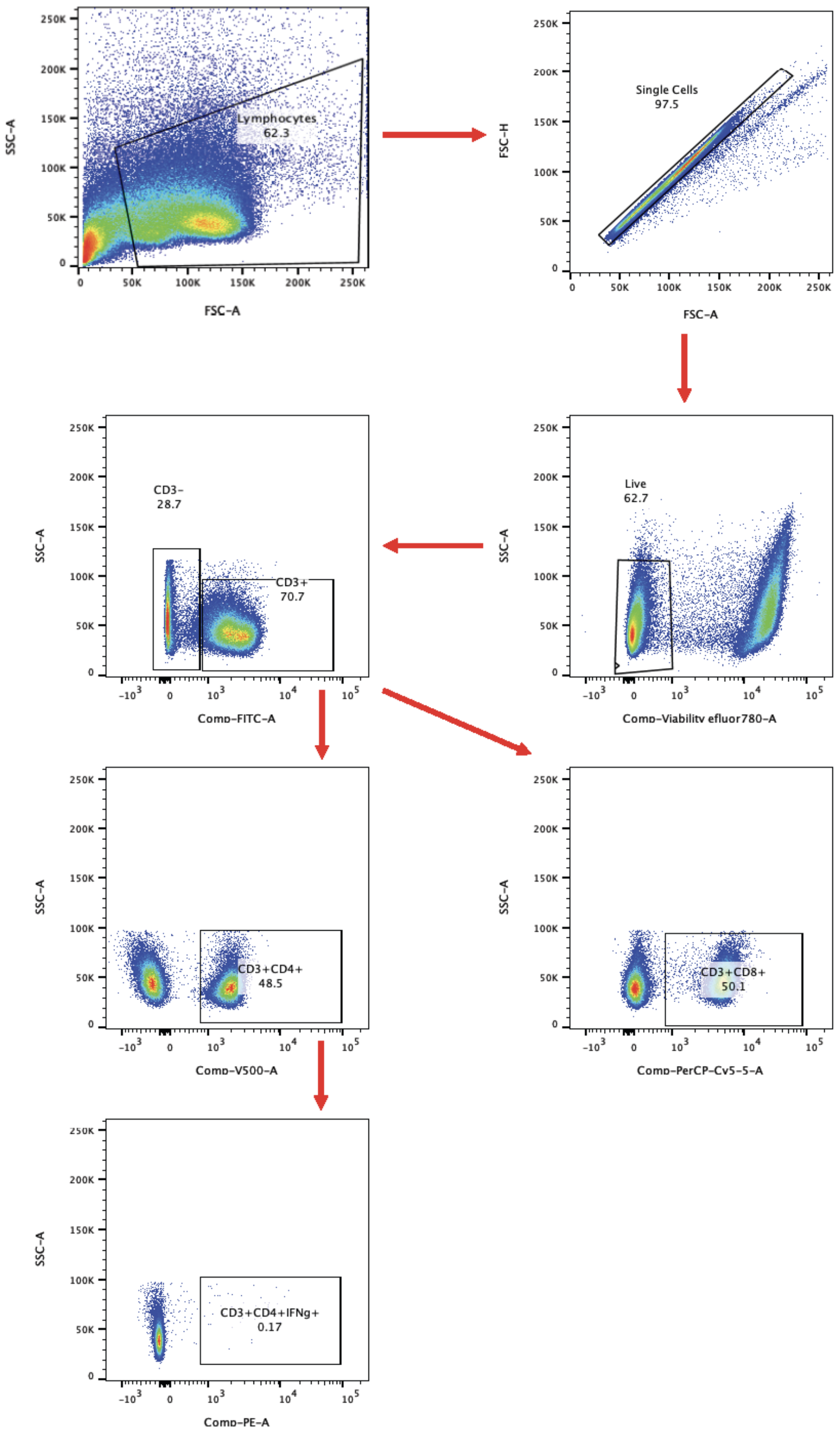

Supplemental Figure 4. Flow cytometry gating strategy. To identify $CD4^+$ and $CD8^+$ T cells expressing IFNγ, we first gated to exclude debris, cell clusters, and dead cells. After gating for single cells and live cells, T cell subsets were identified from the $CD3^+$ population and $IFN\gamma^+$ cells were identified in each population.

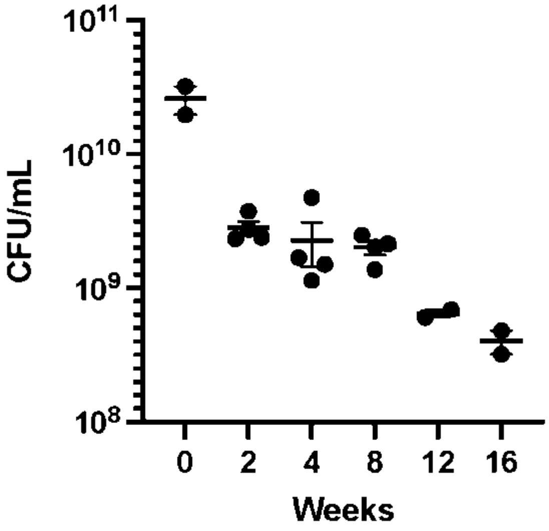

Supplemental Figure 5. Viability of lyophilized NHC over time. Recombinant YS1646 strains were freeze-dried and stored at room temperature (RT) for 16 weeks. Samples were resuspended and grown on LB plates to assess CFU counts and calculate concentrations. The y-axis is expressed on a log scale. Each time point consisted of either 2 or 4 samples.

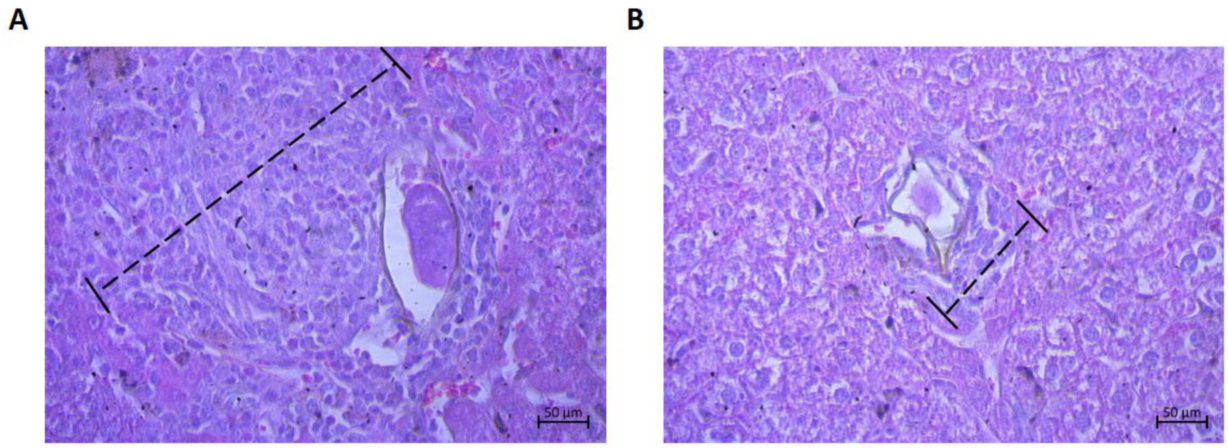

Supplemental Figure 6. Histopathological staining of hepatic sections. Representative images of H&E staining of liver egg granulomas in control mice (A) and the multimodal vaccination group (B). Scale is set to 50 μm.

Supplemental Table 1. Parasitic Burden.

Vaccine Group	Worm Count	% Female Worms	Hepatic Egg Count	Intestinal Egg Count
PBS	51.4 ± 2.2	51.3 ± 1.5	15,974 ± 984.7	12,766 ± 804.3
WT + rCatB	38.7 ± 3.8	49.8 ± 1.3	14,041 ± 259.5	10,331 ± 547.4
NHC	24.8 ± 2.0	38.8 ± 2.4***	7,292 ± 658.2	6,185 ± 353.9
NHC + rCatB	10.1 ± 0.7****	32.6 ± 1.9****	3,965 ± 259.5****	2,796 ± 250.5****

Summary of parasitic burden for each study group. Each experiment was performed twice. Data are shown as mean ± SEM. Statistical significance was determined by one-way ANOVA with Tukey's multiple comparisons (****P* < 0.001, *****P* < 0.0001 compared to the PBS group).
