# Supplementary material for: Salmonella Typhimurium expressing chromosomally integrated Schistosoma mansoni Cathepsin B protects against schistosomiasis in mice
Source: NPJ Vaccines. 2023 Feb 27;8:27. doi: 10.1038/s41541-023-00599-w (PMC9969381; doi:10.1038/s41541-023-00599-w)
Supplement: Supplementary file 2 — REPORTING SUMMARY [file 41541_2023_599_MOESM2_ESM.pdf]

nature portfolio

Corresponding author(s): Momar Ndao

Last updated by author(s): Nov 3, 2022

Reporting Summary

Nature Portfolio wishes to improve the reproducibility of the work that we publish. This form provides structure for consistency and transparency in reporting. For further information on Nature Portfolio policies, see our Editorial Policies and the Editorial Policy Checklist.

Statistics

For all statistical analyses, confirm that the following items are present in the figure legend, table legend, main text, or Methods section.

n/a	Confirmed	
☐	☒	The exact sample size (n) for each experimental group/condition, given as a discrete number and unit of measurement
☐	☒	A statement on whether measurements were taken from distinct samples or whether the same sample was measured repeatedly
☐	☒	The statistical test(s) used AND whether they are one- or two-sided<br>*Only common tests should be described solely by name; describe more complex techniques in the Methods section.*
☒	☐	A description of all covariates tested
☐	☒	A description of any assumptions or corrections, such as tests of normality and adjustment for multiple comparisons
☐	☒	A full description of the statistical parameters including central tendency (e.g. means) or other basic estimates (e.g. regression coefficient) AND variation (e.g. standard deviation) or associated estimates of uncertainty (e.g. confidence intervals)
☐	☒	For null hypothesis testing, the test statistic (e.g. F, t, r) with confidence intervals, effect sizes, degrees of freedom and P value noted<br>*Give P values as exact values whenever suitable.*
☒	☐	For Bayesian analysis, information on the choice of priors and Markov chain Monte Carlo settings
☒	☐	For hierarchical and complex designs, identification of the appropriate level for tests and full reporting of outcomes
☒	☐	Estimates of effect sizes (e.g. Cohen's d, Pearson's r), indicating how they were calculated

Our web collection on statistics for biologists contains articles on many of the points above.

Software and code

Policy information about availability of computer code

Data collection	Flow cytometry data was collected on a BD LSRFortessa or BD LSRFortessa X20 flow cytometer (BD Biosciences)<br>Antibody ELISA plates were read at 450 nm on an EL800 microplate reader (Biotek)<br>Cytokine multiplex ELISA plates were read using a Q-View imager (Quansys Biosciences)
Data analysis	Flow cytometry data was analysed using FlowJo software vl0 (Treestar, Ashland)<br>Cytokine multiplex ELISA data were analyzed using Q-View software (Quansys Biosciences)<br>Statistical analyses were conducted using Graph pad Prism v9.2 .0 software.

For manuscripts utilizing custom algorithms or software that are central to the research but not yet described in published literature, software must be made available to editors and reviewers. We strongly encourage code deposition in a community repository (e.g. GitHub). See the Nature Portfolio guidelines for submitting code & software for further information.

Data

Policy information about availability of data

All manuscripts must include a data availability statement. This statement should provide the following information, where applicable:

- Accession codes, unique identifiers, or web links for publicly available datasets
- A description of any restrictions on data availability
- For clinical datasets or third party data, please ensure that the statement adheres to our policy

Data generated in the current study are available from the corresponding author.

Human research participants

Policy information about studies involving human research participants and Sex and Gender in Research.

Reporting on sex and gender	n/a
Population characteristics	n/a
Recruitment	n/a
Ethics oversight	n/a

Note that full information on the approval of the study protocol must also be provided in the manuscript.

Field-specific reporting

Please select the one below that is the best fit for your research. If you are not sure, read the appropriate sections before making your selection.

☒ Life sciences ☐ Behavioural & social sciences ☐ Ecological, evolutionary & environmental sciences

For a reference copy of the document with all sections, see nature.com/documents/nr-reporting-summary-flat.pdf

Life sciences study design

All studies must disclose on these points even when the disclosure is negative.

Sample size	Group sizes were selected based on experience with similar previous studies. No sample size calculations were performed. For each portion of the study, we conducted 2 independent experiments with n = 5 mice per group each.
Data exclusions	No data were excluded from the analyses.
Replication	All mouse experiments were repeated twice to ensure reproducibility. For ELISA, all samples were run in duplicate. All attempts at replication were successful.
Randomization	All mice were randomly assigned to vaccination groups.
Blinding	Investigators were blinded for parasite challenge mouse experiments to avoid any bias when determining parasite burden.

Reporting for specific materials, systems and methods

We require information from authors about some types of materials, experimental systems and methods used in many studies. Here, indicate whether each material, system or method listed is relevant to your study. If you are not sure if a list item applies to your research, read the appropriate section before selecting a response.

Materials & experimental systems

n/a	Involved in the study
☐	☒ Antibodies
☐	☒ Eukaryotic cell lines
☒	☐ Palaeontology and archaeology
☐	☒ Animals and other organisms
☒	☐ Clinical data
☒	☐ Dual use research of concern

Methods

n/a	Involved in the study
☒	☐ ChIP-seq
☐	☒ Flow cytometry
☒	☐ MRI-based neuroimaging

Antibodies

Antibodies used	CD16/CD32 (Fe block) - BO 553142<br>CD3 FITC (clone 145-2Cll) - eBioscience (Thermo Fisher) 11-0031-86<br>CD4 V500 (clone RM4-5) - BO 560782<br>CD8 PerCP-Cy5.5 (clone 53-6.7) - BO 551162<br>CD44 BUV395 (clone IM7) - BO 740215<br>CD62L BUV737 (clone MEL-14) - BO 612-833<br>IFNg PE (clone XMGl.2) - BO 562020
Validation	All antibodies were validated by the manufacturer and were titrated prior to use.

Eukaryotic cell lines

Policy information about cell lines and Sex and Gender in Research

Cell line source(s)	RAW 264.7 - ATCC TIB-71
Authentication	Authenticated by vendor.
Mycoplasma contamination	ATCC cell lines are guaranteed to be mycoplasma negative and no mycoplasma contamination was detected.
Commonly misidentified lines (See ICLAC register)	n/a

Animals and other research organisms

Policy information about studies involving animals; ARRIVE guidelines recommended for reporting animal research, and Sex and Gender in Research

Laboratory animals	Female 6–8-week-old C57BL/6 mice were obtained from Charles River.
Wild animals	The study did not involve wild animals.
Reporting on sex	The study involved female C57BL/6 mice. Sex was not considered during the design or analysis of the experiments.
Field-collected samples	The study did not involve samples collected from the field.
Ethics oversight	All animal procedures were conducted in accordance with Institutional Animal Care and Use Guidelines and were approved by the Animal Care and Use Committee at McGill University (Animal Use Protocol 7625) as well as the Canadian Council on Animal Care.

Note that full information on the approval of the study protocol must also be provided in the manuscript.

Flow Cytometry

Plots

Confirm that:

☒ The axis labels state the marker and fluorochrome used (e.g. CD4-FITC).

☒ The axis scales are clearly visible. Include numbers along axes only for bottom left plot of group (a 'group' is an analysis of identical markers).

☒ All plots are contour plots with outliers or pseudocolor plots.

☒ A numerical value for number of cells or percentage (with statistics) is provided.

Methodology

Sample preparation	Splenocytes were isolated as previously described (Ricciardi 2016; Hassan, 2019; Hendin, 2022).
Instrument	All flow cytometry was conducted using a BD LSRFortessa X20.
Software	Data was analyzed using FlowJo software version 10.8.1 (Treestar, Ashland).
Cell population abundance	No sorting was performed.
Gating strategy	Gating strategies are supplied in supplemental figures.

☒ Tick this box to confirm that a figure exemplifying the gating strategy is provided in the Supplementary Information.
